# Clinically Relevant Germline Variants in Children With Nonmedullary Thyroid Cancer

**DOI:** 10.1210/clinem/dgae107

**Published:** 2024-02-28

**Authors:** Karin van der Tuin, Dina Ruano, Jeroen Knijnenburg, Rob B van der Luijt, Hans Morreau, Thera P Links, Frederik J Hes, Dutch Pediatric Thyroid Cancer Consortium, Mariëlle S Klein Hesselink, Marloes Nies, Gianni Bocca, Adrienne H Brouwers, Johannes G M Burgerhof, Eveline W C M van Dam, Bas Havekes, Marry M van den Heuvel-Eibrink, Eleonora P M Corssmit, Leontien C M Kremer, Romana T Netea-Maier, Helena J H van der Pal, Robin P Peeters, John T M Plukker, Cécile M Ronckers, Hanneke M van Santen, Jan W A Smit, Thera P Links, Wim J E Tissing, Karin van der Tuin, Frederik J Hes, Evert F van Velsen, Rachel S van Leeuwaarde

**Affiliations:** Department of Clinical Genetics, Leiden University Medical Center, 2333 ZA Leiden, The Netherlands; Department of Pathology, Leiden University Medical Center, 2333 ZA Leiden, The Netherlands; Department of Clinical Genetics, Leiden University Medical Center, 2333 ZA Leiden, The Netherlands; Department of Clinical Genetics, Leiden University Medical Center, 2333 ZA Leiden, The Netherlands; Department of Pathology, Leiden University Medical Center, 2333 ZA Leiden, The Netherlands; Department of Endocrinology, Internal Medicine, University of Groningen, University Medical Center Groningen, 9713 GZ Groningen, The Netherlands; Department of Clinical Genetics, Leiden University Medical Center, 2333 ZA Leiden, The Netherlands; Clinical Sciences, Research Group Reproduction and Genetics, Center for Medical Genetics, Vrije Universiteit Brussel (VUB), Universitair Ziekenhuis Brussel (UZ Brussel), 1090 Jette Brussels, Belgium

**Keywords:** nonmedullary thyroid cancer, pediatrics, genetics, heritability

## Abstract

**Context:**

The underlying genetic cause of nonmedullary thyroid cancer (NMTC) in children is often unknown, hampering both predictive testing of family members and preventive clinical management.

**Objective:**

Our objectives were to investigated the potential heritability in the largest childhood NMTC cohort that has been genotyped to date.

**Methods:**

Nationwide retrospective cohort study in tertiary referral centers. In total, 97 patients diagnosed with pediatric NMTC between 1970 and 2020 were included in this study. Patients underwent germline whole genome sequencing. The main outcome measures were mutation detection yield in (1) clinically relevant tumor predisposition genes and (2) genes previously associated with NMTC.

**Results:**

In total, 13 of 97 patients (13%) carried a germline (likely) pathogenic variant in a well-known tumor predisposition gene: *APC* (n = 1), *BRCA2* (n = 2), *CHEK2* (n = 4), *DICER1* (n = 4), *HOXB13* (n = 1), and *MITF* (n = 1). In addition, 1 patient was diagnosed with Pendred syndrome (*SLC26A4*) and 9 variants of high interest were found in other NMTC candidate susceptibility genes.

**Conclusion:**

The reported prevalence (13%) of germline variants in well-known tumor predisposing genes and the added value of a revised personal/family history and histology led us to recommend genetic counseling for all patients with childhood NMTC. The detected tumor predisposition syndromes are associated with a risk for second cancers which necessitates additional surveillance of the index patients and presymptomatic genetic testing of at risk family members.

Papillary and follicular thyroid carcinomas are collectively referred to as nonmedullary thyroid carcinomas (NMTCs) and represent more than 90% of all thyroid cancers (1). While NMTC has a generally good prognosis in children compared with adults, it presents at a more advanced stage and is associated with relatively high rates of recurrence (7%), persistent disease (8%), and postoperative complications (>30%) (2). Global age-standardized incidence rates of thyroid cancer among children and adolescents aged 0-19 years ranged from 0.4 to 13.4 cancers per 1 million person-years in 2008-12 (3).

Recognizable features of a genetic predisposition to cancer are the development of 1 or more malignancies at an earlier than expected age and/or familiar clustering of cancer. Although NMTC meets these characteristics (eg, 16% diagnosed below 35 years, increased risk for a second primary tumor and a 3- to 10-fold increased risk in relatives) (4, 5), most NMTC cases remain genetically unexplained. Besides important clinical implications for the index patient, identification of a causative germline variant also facilitates cascade testing and surveillance of relatives, allowing early identification of (pre)malignant conditions.

Familial nonmedullary thyroid cancer constitutes 3% to 9% of all thyroid cancers and is recognized as a distinct clinical entity, with heritability partially attributable to tumor predisposition syndromes such as *PTEN* hamartoma tumor syndrome, Carney complex, familial adenomatous polyposis, *DICER1* syndrome, and Werner syndrome (6). In addition, genome-wide association studies and family-based case series have suggested a variety of possible associated genes (eg, *DIRC3*, *FOXE1*, *NRG1*, *SMAD3*, *SRGAP1*, *SRRM2*, and *TITF-1/NKX2.1*) (7, 8). Furthermore, recent studies indicate that NMTC heritability may be partly polygenic (9).

As no clear guidelines for genetic testing for NMTC are currently available, an important unanswered question is whether all pediatric patients should undergo genetic testing and, if so, which type of genetic test (eg, single gene/gene panel/whole exome or genome sequencing) should be performed. Due to difficulties recruiting pediatric NMTC cases, previous genomic studies have predominantly focused on adult series and/or limited candidate gene approaches, mainly in highly selected and relatively small cohorts. Using DNA gathered from patient recruitment spanning 50 years, we systematically and comprehensively analyzed whole genome sequencing data from 97 childhood cases of NMTC, with the aim of determining the mutation detection yield in (1) clinically relevant tumor predisposition genes, and (2) genes previously associated with NMTC.

## Materials and Methods

### Study Population, Design, and Ethics Approval

All patients diagnosed with NMTC during childhood (<18 years old) in The Netherlands between 1970 and 2012 (n = 170) were eligible and contacted for inclusion in a nationwide study to evaluate the presentation, complications, and long-term outcome in patients with pediatric thyroid cancer (2). A total of 105 patients gave informed consent and were included, of whom 81 also agreed to genetic testing (those aged above 18 years). Personal history and histology data were collected from the patient files and family history was based on a self-reported questionnaire. During the aforementioned period children with NMTC but without clear features of a specific tumor predisposition syndrome were not automatically offered genetic testing. Three patients were excluded due to detection of a known tumor predisposition syndrome during diagnostic workup (2 *DICER1* and 1 *PTEN*). Over the period 2013-2020, an additional 19 patients consented to genetic testing under the same conditions as the initial cohort. In total, 97 patients provided informed consent for the collection of clinical, histological, and molecular data.

To describe the clinical characteristics, the mean was calculated. Continuous variables were analyzed using an independent sample t-test. Dichotomous variables were compared using the chi-squared test. Statistical significance was set at *P* < .05; analyses were conducted using SPSS 23.0 (SPSS, Chicago, IL).

The current study was approved by the Leiden University Medical Center ethics committee (LUMC B17.042). Participants were informed by their attending physician of causal and/or clear “actionable” pathogenic (P)/likely pathogenic (LP) variants, as indicated in the informed consent. Before reporting, findings were discussed by a multidisciplinary team, in line with diagnostic practice of the LUMC Department of Clinical Genetics and ACMG recommendations for reporting secondary findings.

### Germline DNA Sequencing

Following quality control and library preparation (TruSeq PCR-Free library), whole genome sequencing was performed by Macrogen (Seoul, Republic of Korea) using the Illumina HighSeq X Ten (2× 150 bp). DNA fragments were mapped to hg19 using the Isaac aligner. Full details of sample preparation and sequencing are provided elsewhere (Supplementary Appendix 1 (10)). The whole genome sequencing data statistics are shown elsewhere (Table S1 and Fig. S1 (10)).

### Tumor Predisposition Gene Selection for Analysis

In total, 780 genes were selected for analysis on the basis of available literature and divided into 4 nonoverlapping gene panels (see Table S2) (10):

1. The first gene panel (autosomal dominant tumor predisposition genes) consisted of 66 genes with well-established associations with monogenic cancer risk (see full details in Table S3) (10). This gene list primarily consists of “actionable” genes with a significant cancer risk substantiated by literature evidence and with a potential effect on clinical practice, including treatment choices and detection/prevention opportunities. Nine genes on this list are associated with increased NMTC risk.
2. The second gene panel (autosomal recessive/X-linked tumor predisposition genes) consisted of 38 genes (see full details in Table S4) (10).
3. The third gene panel (NMTC candidate susceptibility genes) consisted of 43 genes associated with NMTC based solely on genome-wide association study or whole exome sequencing family studies (see literature overview in Table S5) (10).
4. The fourth gene panel (other cancer genes) included an additional 633 genes potentially related to cancer (see literature overview in Table S6 (10)). This category is as complete as possible based on current literature and consists of low penetrance tumor predisposition genes, genes associated with neurodevelopmental syndromes and increased cancer risk, tumor suppressor genes, tyrosine kinases genes, and genes selected on the basis of recurrent somatic variants in cancer.

### Data Analysis

All unique coding variants, including single nucleotide variants and small insertions and deletions, were annotated using an in-house pipeline that compared data with population and disease databases (eg, ExAC, ClinSig, and InterVar) and utilized in silico prediction programs (eg, FATHMM, SIFT, Polyphen, MutationTaster, PROVEAN). Variants were initially classified into 3 tiers (high/moderate/low interest) based on population frequency, variant type, predictions of functional impact, and matches to curated variant databases. Our workflow for review of variant pathogenicity is described elsewhere (Supplementary Appendix 1 (10) and summarized in Fig. S2 (10)). Variants of high interest were subjected to manual panel review and classified to 1 of 5 tiers according to ACMG guidelines (11): P, LP, variant of uncertain significance, likely benign, benign.

Copy number variant analysis was performed using NxClinical 5.2 (BioDiscovery, El Segundo, CA, USA) against a reference set (described in detail in Supplementary Appendix 1 (10)). Copy number variants that overlapped with genes from the 4 gene panels were visually inspected for validity and, where applicable, validated using a second technique.

### Molecular Tumor Analysis

Molecular tumor analysis (somatic DNA variant and/or gene fusion analyses) was performed, depending on the time period, using a range of different methods according to the specific research question (eg, tumor classification, prognostic forecasting, second-hit analyses). Details can be found elsewhere (Supplementary Appendix 1 (10)).

## Results

### Patient Characteristics

The clinical characteristics of the study population are listed in Table 1. The median age at diagnosis was 15 years (range 7-18 years), with strong female overrepresentation (88% female). Based on histology, 82 patients were diagnosed with papillary thyroid cancer (including various subtypes such as classical, follicular variant, tall cell, and mixed type) and 15 with follicular thyroid cancer. Persistent or recurrent disease was reported in 14 patients. Three patients were diagnosed with cancer and/or received radiotherapy prior to NMTC diagnosis, and 5 patients developed a second primary tumor after NMTC. Mean age at follow-up was 36 years (range 22-68 years).

**Table 1.** Characteristics of the 97 nonmedullary thyroid cancer patients included in this study

Clinical characteristics	Total study population (n = 97)	Cases with P/LP (n = 13)	Case without P/LP (n = 84)	*P* value
**Mean age**				
At diagnosis, years (range)	15 (7-18)	15 (8-18)	15 (7-18)	.858
At last follow-up, years (range)	36 (22-68)	38 (23-63)	36 (22-68)	.461
**Sex**				.582
Male, n = (%)	12 (12)	1 (8)	11 (13)	
Female, n = (%)	85 (88)	12 (92)	73 (87)	
**Histology**				.025
Papillary thyroid carcinoma, n = (%)	81 (84)	9 (70)	72 (86)	
Follicular thyroid carcinoma, n = (%)	15 (15)	3 (23)	12 (14)	
Poorly differentiated thyroid carcinoma, n = (%)	1 (1)	1 (8)	0 (0)	
**History cancer**				
Personal prior NMTC, n = (%)	3 (3)	0 (0)	3 (4)	.489
Personal after NMTC, n = (%)	5 (5)	1 (8)	4 (5)	.657
Family*^a^*, n = (%)	40 (65)	9 (81)	31 (80)	.186
**Outcome**				.669
Remission, n = (%)	63 (65)	10 (77)	53 (63)	
Persistent, n = (%)	7 (7)	1 (8)	6 (7)	
Recurrence, n = (%)	7 (7)	1 (8)	6 (7)	
Unknown, n = (%)	20 (21)	1 (8)	19 (22)	

Abbreviations: NMTC, nonmedullary thyroid carcinoma.

^*a*^Family history known of 62 out of 97 patients, defined here as at least 1 first- and/or 2 second-degree relatives with cancer. Only 2 patients had a self-reported relative with NMTC.

### Germline Variants

In total, 1492 unique rare nonsynonymous single nucleotide variants and small indels were identified in 536 of the 780 genes selected. The results of variant interpretation strategy are listed in Table 2. In summary, 91 variants were classified as high interest, 1039 variants of moderate interest, and 387 variants of low interest. All variants of high and moderate interest are listed elsewhere (Table S7a-d (10)). No validated copy number variants of interest were found among the 780 selected genes.

**Table 2.** Results gene selection and variant classification strategy

	Autosomal dominant	Autosomal recessive/X-linked	NMTC candidate susceptibility	Other cancer genes	Total
Number of genes included	66	38	43	633	780
Unique variants	534	314	361	4442	5651
Unique rare variants (allele frequency <0.01)	263	140	138	1922	2463
**Unique rare nonsynonymous variants**	161	102	93	1161	1517
High interest (%)^a^	15 (9)	8 (8)	12 (13)	56 (5)	91 (6)
Moderate interest (%)^a^	63 (39)	51 (50)	73 (78)	852 (73)	1039 (68)
Low interest (%)^a^	83 (52)	43 (42)	8 (9)	253 (22)	387 (26)

See Table S2 for gene selection. All variants of high and moderate interest are listed in Table S7a-d.

^*a*^Of total unique rare nonsynonymous variants within gene panel.

### Germline Variants in Tumor Predisposition Genes

In total, 13 patients (13%) carried a P/LP variant in a known autosomal dominant tumor predisposition gene: *APC* (n = 1), *BRCA2* (n = 2), *CHEK2* (n = 4), *DICER1* (n = 4), *HOXB13* (n = 1), and *MITF* (n = 1). The variants are listed in Table 3 and the clinical and tumor characteristics of the 13 patients with P/LP variants are summarized in Table 4. The *APC* and *DICER1* variants are considered causal and clinically relevant during childhood. The other P/LP variants are associated with adult-onset tumor predisposition syndromes, of which *BRCA2* and *CHEK2* in particular have relevance in terms of surveillance for the index patients and relatives. Further evidence supporting the final classification of (likely) pathogenic variants can be found elsewhere (Table S8 (10)). No homozygote or compound heterozygote P/LP variants were found among the autosomal recessive tumor predisposition genes.

**Table 3.** Detected (likely) pathogenic clinically relevant germline variants and associated tumor predisposition syndromes

Deteactcted (likely) pathogenic germline variants						Tumor predisposition syndrome				
Case	Gene	Reference sequence	Variant	Variant effect	ACMG class*^a^*	Syndrome	Major associated tumor types	Increased NMTC risk	Penetrance	Inheritance mode
718	*APC*	NM_000038.5	c.2434_2437del, p.(Asp812Ilefs*7)	Frameshift	5	Familial adenomatous polyposis	Colorectal cancer	Y	High	AD
							Hepatoblastoma			
							Desmoid tumor			
514	*BRCA2*	NM_000059.3	c.5946del, p.(Ser1982Argfs*22)	Frameshift	5	Hereditary breast–ovarian cancer	Breast cancer	N	High	AD
							Ovarian cancer			
703			c.516+1G>T, p.?	Splice	5		Prostate cancer			
							Pancreas cancer			
109	*CHEK2*	NM_0007194.3	c.1100del, p.(Thr367Metfs*15)	Frameshift	5		Breast cancer	Y	Moderate	AD
304			c.1100del, p.(Thr367Metfs*15)	Frameshift	5					
408			c.499G>A, p.(Gly167Arg)	Missense	4					
512			c.1037G>A, p.(Arg346His)	Missense	4					
106	*DICER1*	NM_177438.2	c.3300dup, p.(Ser1101Ilefs*3)	Frameshift	5	DICER1 syndrome	Pleuropulmonary blastoma	Y	Moderate	AD
115			c.3300dup, p.(Ser1101Ilefs*3)	Frameshift	5		Cystic nephroma			
504			c.2270T>, p.(Leu757*^a^*)	Nonsense	5		Ovarian sex cord tumor			
509			c.2256+1G>C, p.?	Splice	5		Thyroid neoplasia			
709	*HOXB13*	NM_006361.5	c.251G>A, p.(Gly84Glu)	Missense	5		Prostate cancer	N	Moderate	AD
417	*MITF*	NM_001354604	c.1273G>A, p.(Glu425Lys)	Missense	5		Melanoma	N	Moderate	AD

Abbreviations: ACMG, American College of Medical Genetics; AD, autosomal dominant; AR, autosomal recessive; NMTC, nonmedullary thyroid cancer.

^*a*^See Table S8 for the evidence supporting the variant classification, including recent functional data of CHEK2 variants.

**Table 4.** Clinical and tumor characteristic of the 14 patients diagnosed with a tumor predisposition syndrome

Clinical characteristic									Tumor characteristic			
Case	Gene	Gender	Age Dx NMTC	Personal history	Follow-up	Family history on form	Family history after genetic counseling	Variant segregation family	Histology report	Histology revision*^a^*	Molecular test	Somatic variant
718	*APC*	F	16	N	Polyps colon	N	NA	Y (de novo)	PTC	CMTC	1, 2	NCOA4/RET fusion
514	*BRCA2*	M	15	N		Y (B, 1st, B/O, 2nd)	Same as form	N (paternal)	PTC	cPTC	3, 4,5	NCOA4/RET fusion
703		F	9	N		Y (B, 1st)	Same as form	NA	cPTC	cPTC	1, 2, 4	NCOA4/RET fusion
304	*CHEK2*	F	16	N		N	NA	NA	cPTC	NA	1, 2	No
109		F	16	N		N	NA	NA	PTC	NA	1, 2	*BRAF* ^V600E^
408		F	17	N		Y (B, 2nd)	NA	NA	PTC	NA	NA	NA
512		F	18	N		Y (B, 2nd)	NA	NA	FVPTC	NA	NA	NA
115*^a^*	*DICER1*	F	15	N		N	Y (TC, 2nd)	Y	PTC	NA	NA	NA
106*^a^*		F	14	N		N	Y (TC, 2nd)	Y	FTC	NA	NA	NA
504		F	15	N		N	Y (lung cyst, 1st)	Y	FTC	Insular TC	NA	NA
509		F	14	N		N	Y (MNG, 1st)	Y	PTC	MNG+PDTC	3, 5	*DICER1* (E1813Q), *TP53* (c.1027_1033del)
709	*HOXB13*	F	18	N		N	NA	NA	FVPTC	NA	NA	NA
417	*MITF*	F	16	N		NA	NA	NA	PTC	NA	NA	NA

Abbreviations: 1, PCR BRAF; 2 RET/PTC FISH; 3, cancer hotspot panel; 4, HRD panel; 5, gene fusion panel. B, breast cancer; (c) PTC, (classic) papillary thyroid carcinoma; CMTC, cribriform morular thyroid carcinoma; Dx, diagnosis; F, female; FTC, follicular thyroid carcinoma; FVPTC, follicular variant papillary thyroid carcinoma; M, male; MNG, multinodular goiter, Y, yes; N, no; NA, not available; O, ovarian cancer; TC, thyroid cancer.

^*a*^Second degree cousins become clear after genetic analysis.

### NMTC Candidate Susceptibility Genes and Other Cancer Genes

Eleven variants of high interest were identified in previously reported NMTC candidate susceptibility genes (listed in Table S7c (10)). One patient was diagnosed with Pendred syndrome (associated with congenital deafness, multinodular goiter, and rarely NMTC). Furthermore, 1 previously reported likely pathogenic *FOXE1* variant was found, as well as rare heterozygous variants in *DUOX2*, *HABP2*, *PARP4*, *SERPINA1*, and *SRRM2.* In the category “other cancer genes,” 48 variants of high interest were detected (listed in Table S7d (10)).

### Correlation Between Genotype and Phenotype

In general, the clinical characteristic of patients with or without P/LP did not show significantly differences, except for histological subtype (see Table 1). The majority of germline P/LP variant carriers had no reported personal or family history related to the detected tumor predisposition syndrome. However, after reporting the detected variant to the patients, a more detailed family history revealed benign lung cysts and/or thyroid neoplasia in all *DICER1* pedigrees. Variant segregation analysis in the DICER1 families confirmed that all affected family members were carrying the germline *DICER1* variant. The patient with a pathogenic *APC* variant developed multiple colonic polyps after diagnosis of NMTC. The *APC* variant was confirmed to be de novo, as expected based on the negative family history. We cannot state with certainty that the P/LP *BRCA2*, *CHEK2*, *HOXB13*, and *MITF* variants contributed to the patient's cancer, these might also be secondary findings. In 2 out of 6 patients with P/LP *CHEK2* or *BRCA2* variants, family history included breast cancer, but none met the current guidelines for genetic testing. In case 514 the maternal family history was positive for breast and ovarian cancer, however the *BRCA2* variant was confirmed to be inherited from the father (small family with few female relatives). Segregation analysis in *HOXB13-* and *MITF* case was not (yet) performed.

### Correlation Between Genotype and Tumor Characteristics

In none of the pathology reports stated any specific clues for genetic predisposition. However, re-evaluation of available tumors revealed in a number of cases subtle histological and molecular clues for specific tumor predisposition syndromes. The 2 available *DICER1*-related tumors showed diffuse nodular hyperplasia with multiple, discrete, well-circumscribed and occasionally encapsulated nodules (Fig. S3 (10)). In addition, a somatic second-hit *DICER1* variant affecting the RNase IIIb domain was found in a tumor following comprehensive molecular testing (12). Due to morules and a cribriform growth pattern, the *APC*-related tumor was reclassified as a cribriform morular thyroid carcinoma. We found this was not stated as such in the initial pathology report. Additional immunohistochemical staining showed nuclear β-catenin staining related to the permanent activation of the Wnt pathway (Fig. S4 (10)). An oncogenic *RET* rearrangement, a known driver of cribriform morular thyroid carcinoma, was also found in this tumor. Remarkably, the tumors from the patients with a germline pathogenic *BRCA2* variant both revealed an oncogenic *RET* rearrangement, while showing no conclusive evidence for loss of heterozygosity of *BRCA2* nor a second (somatic) variant.

## Discussion

Genetic screening of a large, unbiased pediatric NMTC cohort detected a relatively high prevalence (13%) of P/LP germline variants in well-known tumor predisposing genes. The majority of the tumor predisposition syndromes detected by this study are associated with a risk for second cancers for which additional surveillance is recommended. Additionally, these syndromes all show autosomal dominant patterns of inheritance that enable presymptomatic genetic testing of at risk family members.

The detection yield in this series (13%) is higher than in previously reported studies of other solid cancers in childhood. Approximately 6% of the childhood cancer survivors from St. Jude Lifetime Cohort Study carried P/LP germline variants in a cancer predisposition gene (175 out of 3.006 patients) (13). In a further multicenter international study, P/LP germline variants were identified in 7.6% of childhood cancer patients (69 out of 914 individual patients) (14). Correcting for the relative incidence of cancer types as a whole, the authors estimated that approximately 6% of all childhood cancer patients may carry a causative germline variant. In noncancer controls, previous studies showed P/LP germline variants in cancer genes listed by the ACMG in approximately 0.8% to 1.1% (eg, 1000 Genome project) (15, 16).

### Double-Strand Break Repair in Thyroid Cancer

This study found an unexpectedly high prevalence of P/LP germline variants associated with adult-onset tumor predisposition syndromes, and around half of these syndromes were not included in the initial differential diagnosis. In particular, P/LP variants in genes related to the homologous recombination (HR) pathway were overrepresented (2 *BRCA2* and 4 *CHEK2* in 97 cases [total 6%]). As only 12 pediatric patients with NMTC were included in The Cancer Genome Atlas, no substantial validation cohort is available. In the adult Cancer Genome Atlas NMTC cohort 7 patients (1.4%) were identified with a germline P/LP variant in the HR genes: *ATM* (n = 2), *CHEK2* (n = 1), and *BRCA2* (n = 4) (17).

Although we cannot state with certainty that all P/LP variants contributed to the patient's cancer, several lines of evidence might support a link between NMTC and variants affecting DNA double-strand break repair. From a functional and tumor genomics perspective, ionizing radiation is a strong environmental risk factor for NMTC (18) and radiation-induced double-strand breaks are repaired via this pathway (19). Similarly, accidents at nuclear reactors dramatically increased the incidence of NMTC, especially in exposed young children (20). Furthermore, in the absence of significant radiation exposure, somatic oncogenic rearrangement—an event that requires double-strand breaks—is a common driver in NMTC and is even more frequent in childhood NMTC than in adult disease (21, 22). None of the patients with a germline P/LP *BRCA2* or *CHEK2* variant in our series had any history of radiation. Unfortunately, not all tumor material could be reliably tested for second hits or indicative HRD chromosomal copy number profiles. However, even in a state of BRCA haploinsufficiency without second hit, there is currently ample discussion that in certain instances BRCA haploinsufficiency might add to tumorigenesis (23).

In terms of population genomics, the bidirectional increased risk of NMTC and breast cancer suggests a common etiology (24, 25). This is supported by the significantly increased frequency of pathogenic *CHEK2* variants among individuals with a history of both thyroid and breast cancer compared to breast cancer alone (26). Furthermore, NMTC risk is higher in *CHEK2* mutation carriers than in healthy controls (OR 2.8-5.7) (27, 28), but not in *BRCA1* and *BRCA2* mutation carriers (OR 0.6-4.3 and 0.1-3.1, respectively) (29). In addition, *BRCA2*, *CHEK2*, and *ATM* polymorphisms are found more frequently in patients with familial nonmedullary thyroid cancer than in patients with sporadic NMTC and/or healthy controls (30-32). Finally, we also found 3 additional truncating variants in HR pathway genes in our cohort (*ATR*, *FAN1*, and *RAD50*). Further studies are now needed to determine whether these germline P/LP variants, and DNA double-strand break repair more generally, play a role in the development of pediatric NMTC.

### Clinical Genetic Counseling and Testing

Although clinical phenotype, family history, histology, and somatic variants are all important factors in the recognition of an underlying genetic syndrome, around 50% of the clinically relevant P/LP variants identified in this study were nevertheless absent from our initial differential diagnosis based on the patient files. In particular, the recognition of associated benign lesions and rare histological subtypes are challenging for unexperienced physicians. Therefore, children with NMTC should be cared for by teams of experienced physicians including, not only high-volume thyroid surgeons, but also experts in (molecular) pathology, nuclear medicine, endocrinology and clinical genetics (33). The combination of a relatively high prevalence of P/LP variants in pediatric NMTC and the associated clinical impact found in this study now justifies recommending genetic counseling in all cases of childhood NMTC.

This study suggests that broad genetic testing promotes identification of tumor predisposition syndromes that might otherwise be missed, when using single/limited gene testing based solely on the initial differential diagnosis. The obvious drawback of large-scale gene analysis is the complex interpretation of results, including an increased probability of detecting variants of uncertain significance and secondary findings. Pre-test genetic counseling, including establishing patient's and parents personal preferences and tolerance regarding ambiguous or secondary findings, should play a crucial role when selecting a preferred sequencing modality.

Use of radioactive iodine for NMTC treatment increased through 2009, especially in young patients (2). However, in addition to providing limited to no survival benefit for localized NMTC, radioactive iodine is associated with increased risks of short-term and long-term adverse outcomes, including secondary primary malignancies (34, 35). The presence of germline P/LP variants in tumor predisposition genes may influence treatment choices in low-risk tumors.

The main strength of the current study was the use of state of the-art genetic testing in a large, clinically well-defined pediatric NMTC series, the largest of its kind analyzed to date (n = 97). Furthermore, potential selection bias was minimized by approaching all children diagnosed with NMTC in The Netherlands within a defined period (1970-2020). Details on case ascertainment were published (2). Nevertheless, this study had certain limitations which suggest that the mutation detection yield might even be an underestimate. First, manually classified variants of “high interest” and “moderate interest” were confined to genes associated with clinically relevant tumor predisposition syndromes, while additional novel cancer-associated genes, structural variations, changes in noncoding regions, and epigenetic modifications may be recognized in the future. Second, even after expert panel review many variants remain of “uncertain significance.” Molecular testing of tumors and functional testing might have augmented evidence of pathogenicity in some cases but could not be performed in most of the cases. Third, mosaic mutations may have been missed due to insufficient sequencing depth. And finally, the parents or relatives of the patients in our cohort were not studied and hence could not be assessed regarding whether variants were either de novo or segregated with a (cancer) phenotype in family members.

In conclusion, the reported prevalence of clinically relevant P/LP germline variants in well-known tumor predisposition genes and added value of revised personal/family history and histology led us to recommend genetic counseling for all patients with childhood NMTC.

## Data Availability

Most original data generated and analyzed during this study are included in the supplemental data (https://doi.org/10.17026/dans-xrs-2zxp). Some data are not publicly available but are available from the corresponding author on reasonable request.

## Abbreviations

HR: homologous recombination
LP: likely pathogenic
NMTC: nonmedullary thyroid carcinoma
P: pathogenic

## References

[1] Shapiro NL , Bhattacharyya N. Population-based outcomes for pediatric thyroid carcinoma. Laryngoscope. 2005;115(2):337‐340. 15689762 10.1097/01.mlg.0000154743.71184.09

[2] Klein Hesselink MS , Nies M, Bocca G, et al Pediatric differentiated thyroid carcinoma in The Netherlands: a nationwide follow-up study. J Clin Endocrinol Metab. 2016;101(5):2031‐2039. 26963949 10.1210/jc.2015-3290

[3] Vaccarella S , Lortet-Tieulent J, Colombet M, et al Global patterns and trends in incidence and mortality of thyroid cancer in children and adolescents: a population-based study. Lancet Diabetes Endocrinol. 2021;9(3):144‐152. 33482107 10.1016/S2213-8587(20)30401-0

[4] Goldgar DE , Easton DF, Cannon-Albright LA, Skolnick MH. Systematic population-based assessment of cancer risk in first-degree relatives of cancer probands. J Natl Cancer Inst. 1994;86(21):1600‐1608. 7932824 10.1093/jnci/86.21.1600

[5] Endo M , Liu JB, Dougan M, Lee JS. Incidence of second malignancy in patients with papillary thyroid cancer from surveillance, epidemiology, and End results 13 dataset. J Thyroid Res. 2018;2018:8765369. 30046434 10.1155/2018/8765369 PMC6038658

[6] Zhou J , Singh P, Yin K, et al Non-medullary thyroid cancer susceptibility genes: evidence and disease Spectrum. Ann Surg Oncology. 2021;28(11):6590‐6600. 10.1245/s10434-021-09745-x 33660127

[7] Orois A , Mora M, Halperin I, Oriola J. Familial non medullary thyroid carcinoma: beyond the syndromic forms. Endocrinol Diabetes Nutr (Engl Ed). 2021;68(4):260‐269. 10.1016/j.endien.2020.08.013 34266638

[8] Miasaki FY , Fuziwara CS, Carvalho GA, Kimura ET. Genetic mutations and variants in the susceptibility of familial non-medullary thyroid cancer. Genes (Basel). 2020;11(11):1364. 33218058 10.3390/genes11111364 PMC7698903

[9] Liyanarachchi S , Gudmundsson J, Ferkingstad E, et al Assessing thyroid cancer risk using polygenic risk scores. Proc Natl Acad Sci U S A. 2020;117(11):5997‐6002. 32132206 10.1073/pnas.1919976117 PMC7084156

[10] van der Tuin K , Ruano D, Knijnenburg J, et al Data from: “Clinically Relevant Germline Variants in Children With Nonmedullary Thyroid Cancer”. *DANS*. Deposited 25 October 2023. 10.17026/dans-xrs-2zxp PMC11570363 38415346

[11] Richards S , Aziz N, Bale S, et al Standards and guidelines for the interpretation of sequence variants: a joint consensus recommendation of the American college of medical genetics and genomics and the association for molecular pathology. Genet Med. 2015;17(5):405‐424. 25741868 10.1038/gim.2015.30 PMC4544753

[12] van der Tuin K , de Kock L, Kamping EJ, et al Clinical and molecular characteristics may Alter treatment strategies of thyroid malignancies in DICER1 syndrome. J Clin Endocrinol Metab. 2019;104(2):277‐284. 30260442 10.1210/jc.2018-00774

[13] Wang Z , Wilson CL, Easton J, et al Genetic risk for subsequent neoplasms among long-term survivors of childhood cancer. J Clin Oncol. 2018;36(20):2078‐2087. 29847298 10.1200/JCO.2018.77.8589 PMC6036620

[14] Grobner SN , Worst BC, Weischenfeldt J, et al The landscape of genomic alterations across childhood cancers. Nature. 2018;555(7696):321‐327. 29489754 10.1038/nature25480

[15] Zhang J , Walsh MF, Wu G, et al Germline mutations in predisposition genes in pediatric cancer. N Engl J Med. 2015;373(24):2336‐2346. 26580448 10.1056/NEJMoa1508054 PMC4734119

[16] Kim J , Luo W, Wang M, et al Prevalence of pathogenic/likely pathogenic variants in the 24 cancer genes of the ACMG secondary findings v2.0 list in a large cancer cohort and ethnicity-matched controls. Genome Med. 2018;10(1):99. 30583724 10.1186/s13073-018-0607-5 PMC6305568

[17] Huang KL , Mashl RJ, Wu Y, et al Pathogenic germline variants in 10,389 adult cancers. Cell. 2018;173(2):355‐370.e14. 29625052 10.1016/j.cell.2018.03.039 PMC5949147

[18] Lubin JH , Adams MJ, Shore R, et al Thyroid cancer following childhood low-dose radiation exposure: a pooled analysis of nine cohorts. J Clin Endocrinol Metab. 2017;102(7):2575‐2583. 28323979 10.1210/jc.2016-3529 PMC5505197

[19] Nickoloff JA , Sharma N, Allen CP, et al Roles of homologous recombination in response to ionizing radiation-induced DNA damage. Int J Radiat Biol. 2023;99(6):903‐914. 34283012 10.1080/09553002.2021.1956001 PMC9629169

[20] Miyakawa M . Radiation exposure and the risk of pediatric thyroid cancer. Clin Pediatr Endocrinol. 2014;23(3):73‐82. 25110391 10.1297/cpe.23.73 PMC4125599

[21] Alzahrani AS , Alswailem M, Alswailem AA, et al Genetic alterations in pediatric thyroid cancer using a comprehensive childhood cancer gene panel. J Clin Endocrinol Metab. 2020;105(10):3324‐3334. 10.1210/clinem/dgaa389 32556222

[22] Cordioli MI , Moraes L, Bastos AU, et al Fusion oncogenes are the main genetic events found in sporadic papillary thyroid carcinomas from children. Thyroid. 2017;27(2):182‐188. 27849443 10.1089/thy.2016.0387

[23] Minello A , Carreira A. BRCA1/2 haploinsufficiency: exploring the impact of losing one allele. J Mol Biol. 2024;436(1):168277. 37714298 10.1016/j.jmb.2023.168277

[24] Bolf EL , Sprague BL, Carr FE. A linkage between thyroid and breast cancer: a common etiology? Cancer Epidemiol Biomarkers Prev. 2019;28(4):643‐649. 30541751 10.1158/1055-9965.EPI-18-0877

[25] Kuo JH , Chabot JA, Lee JA. Breast cancer in thyroid cancer survivors: an analysis of the surveillance, epidemiology, and End results-9 database. Surgery. 2016;159(1):23‐29. 26522696 10.1016/j.surg.2015.10.009

[26] Kamihara J , Zhou J, LaDuca H, et al Germline pathogenic variants in cancer risk genes among patients with thyroid cancer and suspected predisposition. Cancer Med. 2022;11(8):1745‐1752. 35174967 10.1002/cam4.4549 PMC9041070

[27] Siołek M , Cybulski C, Gąsior-Perczak D, et al CHEK2 mutations and the risk of papillary thyroid cancer. Int J Cancer. 2015;137(3):548‐552. 25583358 10.1002/ijc.29426

[28] Cybulski C , Górski B, Huzarski T, et al CHEK2 is a multiorgan cancer susceptibility gene. Am J Hum Genet. 2004;75(6):1131‐1135. 15492928 10.1086/426403 PMC1182149

[29] Mersch J , Jackson MA, Park M, et al Cancers associated with BRCA1 and BRCA2 mutations other than breast and ovarian. Cancer. 2015;121(2):269‐275. 25224030 10.1002/cncr.29041 PMC4293332

[30] Yu Y , Dong L, Li D, et al Targeted DNA sequencing detects mutations related to susceptibility among familial non-medullary thyroid cancer. Sci Rep. 2015;5(1):16129. 26530882 10.1038/srep16129 PMC4632085

[31] Bastos HN , Antão MR, Silva SN, et al Association of polymorphisms in genes of the homologous recombination dna repair pathway and thyroid cancer risk. Thyroid. 2009;19(10):1067‐1075. 19772428 10.1089/thy.2009.0099

[32] Wójcicka A , Czetwertyńska M, Świerniak M, et al Variants in the ATM-CHEK2-BRCA1 axis determine genetic predisposition and clinical presentation of papillary thyroid carcinoma. Genes Chromosomes Cancer. 2014;53(6):516‐523. 24599715 10.1002/gcc.22162 PMC4058861

[33] Lebbink CA , Dekker BL, Bocca G, et al New national recommendations for the treatment of pediatric differentiated thyroid carcinoma in the Netherlands. Eur J Endocrinol. 2020;183(4):P11‐P18. 32698145 10.1530/EJE-20-0191

[34] Pasqual E , Schonfeld S, Morton LM, et al Association between radioactive iodine treatment for pediatric and young adulthood differentiated thyroid cancer and risk of second primary malignancies. J Clinical Oncol. 2022;40(13):1439‐1449. 35044839 10.1200/JCO.21.01841 PMC9061144

[35] Clement SC , Peeters RP, Ronckers CM, et al Intermediate and long-term adverse effects of radioiodine therapy for differentiated thyroid carcinoma–a systematic review. Cancer Treat Rev. 2015;41(10):925‐934. 26421813 10.1016/j.ctrv.2015.09.001

